# Validity assessment of self-reported weight and its correction process among Mexican adult women of reproductive age

**DOI:** 10.1371/journal.pone.0235967

**Published:** 2020-07-29

**Authors:** Lucia Hernández-Barrera, Belem Trejo Valdivia, Martha Maria Téllez-Rojo, Simón Barquera, Cinthya Muñoz-Manrique

**Affiliations:** 1 Nutrition and Health Research Center, National Institute of Public Health, Cuernavaca, Morelos, México; 2 Department of Nutrition and Bioprogramming, National Institute of Perinatology, Mexico City, Mexico; University of Mississippi Medical Center, UNITED STATES

## Abstract

**Objective:**

We aimed to evaluate the agreement between self-reported weight (SRW) and measured weight (MW) in adult women of reproductive age, identify characteristics associated with the difference between SRW and MW (DW), and develop a correction procedure for SRW.

**Methods:**

We used data from 3,452 non-pregnant or non-lactating adult women who participated in the Mexican Family Life Survey. Standardized personnel asked women about their weight before measuring weight and height. We conducted a Bland-Altman analysis for agreement and adjusted linear regression models for sociodemographic characteristics.

**Results:**

Mean DW was -0.59±3.21 kg. Difference varied according to Body Mass Index (BMI) and region of residence (p< 0.05). Correction model for log-MW, included the log-SRW, age group (18–34 and 35–49 years), interaction term (age × SRW), log-height, Southern region, and living with a partner. Based on self-reported weight, we observed an overestimation of underweight/normal weight prevalence and an underestimation of overweight or obesity prevalence.

**Conclusion:**

SRW has limitations to be considered as an alternative to MW among women of reproductive age with specific characteristics. Our proposed correction equation may decrease SRW imprecision improving the estimation of overweight and obesity. We suggest that studies consider and adjust the possible bias associated with weight misreporting on health outcomes.

## Introduction

Weight in women of reproductive age is useful to evaluate gestational weight gain, perinatal adverse outcomes, and postpartum weight retention [1]. Weight before and during pregnancy has also been suggested as a risk factor for malnutrition among infants and children [2, 3]. Nevertheless, the availability of measured weight prior to pregnancy is a challenge, since most pregnancies are unplanned and prenatal centers may not have women’s weight history [4, 5]. Therefore, self-reported weight (SRW) is used as a substitute for measured weight (MW) [6].

Several studies from developed and developing countries have evaluated the validity of SRW in different populations. Although findings suggest a good correlation between SRW and MW, this does not imply good agreement between them [7]. Moreover, the misreporting of weight may be differential according to age, Body Mass Index (BMI), pregnancy, or lactation, among other factors [8]. In addition, studies show that agreement, sensitivity, and specificity of BMI vary according to each BMI category when using SRW against MW [9, 10]. However, in Mexico, no studies have proposed a correction equation of SRW in adult women of reproductive age.

SRW has several applications in the health field [11]; therefore, an adjustment of SRW to approximate to MW could contribute not only to estimate the prevalence of overweight and obesity but also to estimate pregestational weight in women of reproductive age.This is relevant because among women of reproductive age, misreporting of weight leads to inadequate BMI classification and gestational weight gain counselling [6]. Since BMI before pregnancy (BMI-p) is associated with multiple maternal and new-born/infant outcomes, an inaccuracy of BMI-p may introduce epidemiological bias and erroneous associations [12].

Therefore, in this study our aims were to evaluate the agreement (concordance) between SRW and MW in Mexican women of reproductive age, to identify sociodemographic characteristics associated with the possible difference between SRW and MW, and to develop a correction procedure for SRW to simulate MW.

## Methods

### Study population

We used data from the Mexican Family Life Survey (MxFLS-3). The MxFLS is a longitudinal and multi-thematic survey with national, urban, rural, and regional representativeness. It has collected data regarding socioeconomic and demographic indicators at an individual, household and community level in three waves: 2002 (MxFLS-1), 2005–2006 (MxFLS-2), and 2009–2012 (MxFLS-3). Additionally, the MxFLS-3 considered information regarding self-reporting and measurements of anthropometric characteristics. The protocol design of each wave was approved by the Institutional Review Board at the National Institute of Public Health in Mexico.

The MxFLS-3 included 8,105 women aged 18–49 years; 4,609 of them were excluded from our research due to pregnancy or lactation (*n* = 1,002), lack of self-reported weight (*n* = 3,373), measured weight (*n* = 230), or measured height (*n* = 4). We also excluded those women for whom the absolute value of the difference between the SRW and MW was higher than ±4 standard deviations (*n* = 44) [13]. The analytic sample considered 3,452 women. An analysis was carried out to evaluate possible biases.

### Procedures

#### Weight measurement

Before the measurements, standardized personnel asked participants about their usual weight. Following the recommended technique [14, 15], weight was recorded using professional electronic scales (Tanita, capacity 150 kg and accuracy of 100 g) and height was measured in duplicate to the nearest 0.1 cm using a stadiometer (Short productions, Olney, Maryland, USA). Measurements were made at each participants’ home; these women were in light clothing and barefoot.

BMI was obtained with both MW and SRW, and measured height. The World Health Organization (WHO) criteria was used to classify women’s weight into four categories: low weight (<18.5 kg/m^2^), normal weight (≥18.5 and <25.0 kg/m^2^), overweight (≥25.0 and <30.0 kg/m^2^), and obesity (≥30.0 kg/m^2^) [16].

#### Sociodemographic characteristics

Questionnaires were administered to collect information regarding reproductive history (number of pregnancies and living children), sociodemographic characteristics (age, marital status, schooling, area of residence, and region of residence), and access to health care services.

#### Age and marital status

Age was categorized into two groups according to risk periods for reproductive and/or perinatal adverse outcomes (18–34 years, 35–49 years). Marital status was classified as women living with or without a partner.

#### Schooling

Years of schooling was categorized into three groups: 1) ≤6 years (elementary school); 2) 7–9 years (secondary school), and 3) >9 years (beyond secondary school).

#### Region and area of residence

To be consistent with the MxFLS, the country was divided into four geographical regions: 1) North: Baja California Sur, Coahuila, Chihuahua, Durango, Nuevo León, Sinaloa, Sonora, and Tamaulipas; 2) Center: Guanajuato, Jalisco, Michoacán, and Morelos; 3) Mexico City; and 4) South: Oaxaca, Puebla, Veracruz, and Yucatan. The Northern region is a more industrialized and developed area than the Southern region is. Areas of residence were further classified as urban (≥2500 inhabitants) or rural (<2500 inhabitants) as defined by the National Institute of Statistics and Geography (Instituto Nacional de Estadística y Geografía, INEGI).

#### Socioeconomic status

A socioeconomic status (SES) for the households was constructed using exploratory factor analysis with categorical variables. Information about household characteristics (i.e. construction materials, floors, walls, roofs, water sources, and types of sewage) and ownership of goods and equipment (i.e. radio, TV set, refrigerator, telephone, and car) were used as components. The main extracted factor explained 36% of the total variance and was stated as SES, which was divided into tertiles to represent low, medium, and high SES.

### Statistical analysis

All analyses were performed using STATA version14. To evaluate a possible selection bias, we compared sociodemographic characteristics between women included and women excluded from the analytical sample. An exploratory data analysis was carried out to identify the distributional behavior of both, SRW and MW, as well as the difference between them. Crude associations were evaluated with respect to age, parity, BMI, region, residence, schooling, and marital status.

#### Comparison between self-reported weight and measured weight

A Bland-Altman approach was used to evaluate the agreement between SRW and MW [17, 18]. Preliminary results indicated the need for a log-transformation of weight variables due to differential variability along values range. Bland-Altman graphics in log scale and derived statistics allowed estimation of percentage of discordant observation between log-weight variables.

#### Prediction of actual weight based on self-reported weight

Multiple linear regression models were used to evaluate the relationship between log-measured weight and log-self-reported weight adjusting for a set of covariates. To evaluate the modifying effect of age, we fitted these models including interaction term(age group × log-SRW). After doing an exploratory analysis, the region variable was limited to only two categories: being from the southern part of the country or from the rest of the country. The final model included log-self-reported weight, log-measured height (meters), interaction term(age group × log-SRW), residence in the South of Mexico, and living with a partner (married or cohabiting) as significant covariates.

## Results

Of the 7,059 women, 3,607 were excluded from the study because they had missing data, thus the analytical sample was 3,452 women of reproductive age. Women excluded from the analysis, compared with women included, were those with less than seven years of schooling (34.7% vs 24.9%), living in a rural area (46.2% vs 42.8%), with lower socioeconomic status (33.2% vs 26.9%), without access to health care services (41.8% vs 33.2%), multipara (79.4% vs 77.3%),and with obesity (33.6% vs 28.7%) (Table 1). Among the 3,452 adult women analyzed, the mean age was 32.5 ± 9.4 years and 55.4% were between 18 and 34 years old. Most women (63.7%) reported being married or living with a partner, having completed at least nine years of schooling (35.5%), and living in an urban area (57.2%).

**Table 1:** Demographic characteristics between women included and women not included in the analysis.

	Total (*n* = 7,059)	Women included in the analysis (*n* = 3,452)	Women not included in the analysis (*n* = 3,607)	*p*-value
**Age (years)**	32.15 ± 9.38	32.51 ± 9.37	31.82 ± 9.39	0.002
**Age groups**				
18–34 years	4,050 (57.4%)	1,911 (55.4%)	2,139 (59.3%)	0.002
35–49 years	3,009 (42.6%)	1,541 (44.6%)	1,468 (40.7%)	
**Years of schooling**				
≤ 6 years	1,934 (29.7%)	826 (24.9%)	1,108 (34.7%)	<0.001
7–9 years	2,352 (36.2%)	1,176 (35.5%)	1,176 (36.9%)	
> 9 years	2,219 (34.1%)	1,314 (39.6%)	905 (28.4%)	
**Marital status**				
Without a partner	2,423 (35.5%)	1,254 (36.3%)	1,169 (34.6%)	0.142
With partner	4,405 (64.5%)	2,198 (63.7%)	2,207 (65.4%)	
**Region of residence**				
North	2,254 (33.0%)	1,178 (34.1%)	1,076 (31.9%)	<0.001
Center	2,810 (41.2%)	1,290 (37.4%)	1,520 (45.0%)	
Mexico City	132 (1.9%)	86 (2.5%)	46 (1.4%)	
South	1,632 (23.9%)	898 (26.0%)	734 (21.7%)	
**Area of Mexico**				
Rural	3,141 (44.5%)	1,476 (42.8%)	1,665 (46.2%)	0.004
Urban	3,916 (55.5%)	1,976 (57.2%)	1,940 (53.8%)	
**Socioeconomic status**				
Low	2,093 (30.1%)	920 (26.9%)	1,173 (33.2%)	<0.001
Medium	2,549 (36.7%)	1,250 (36.6%)	1,299 (36.7%)	
High	2,311 (33.2%)	1,247 (36.5%)	1,064 (30%)	
**Access to health care**				
Yes	4,230 (62.5%)	2,266 (66.8%)	1,964 (58.2%)	<0.001
No	2,535 (34.5%)	1,127 (33.2%)	1,408 (41.8%)	
**Measured Body Mass Index (kg/m**^**2**^**)**				
Low weight	161 (2.4%)	75 (2.2%)	86 (2.6%)	<0.001
Normal weight	2,182 (32.2%)	1,173 (34.0%)	1,009 (30.4%)	
Overweight	2,321 (34.3%)	1,213 (35.1%)	1,108 (11.4%)	
Obesity	2,107 (31.1%)	991 (28.7%)	1,116 (33.6%)	

**Fig 1:**
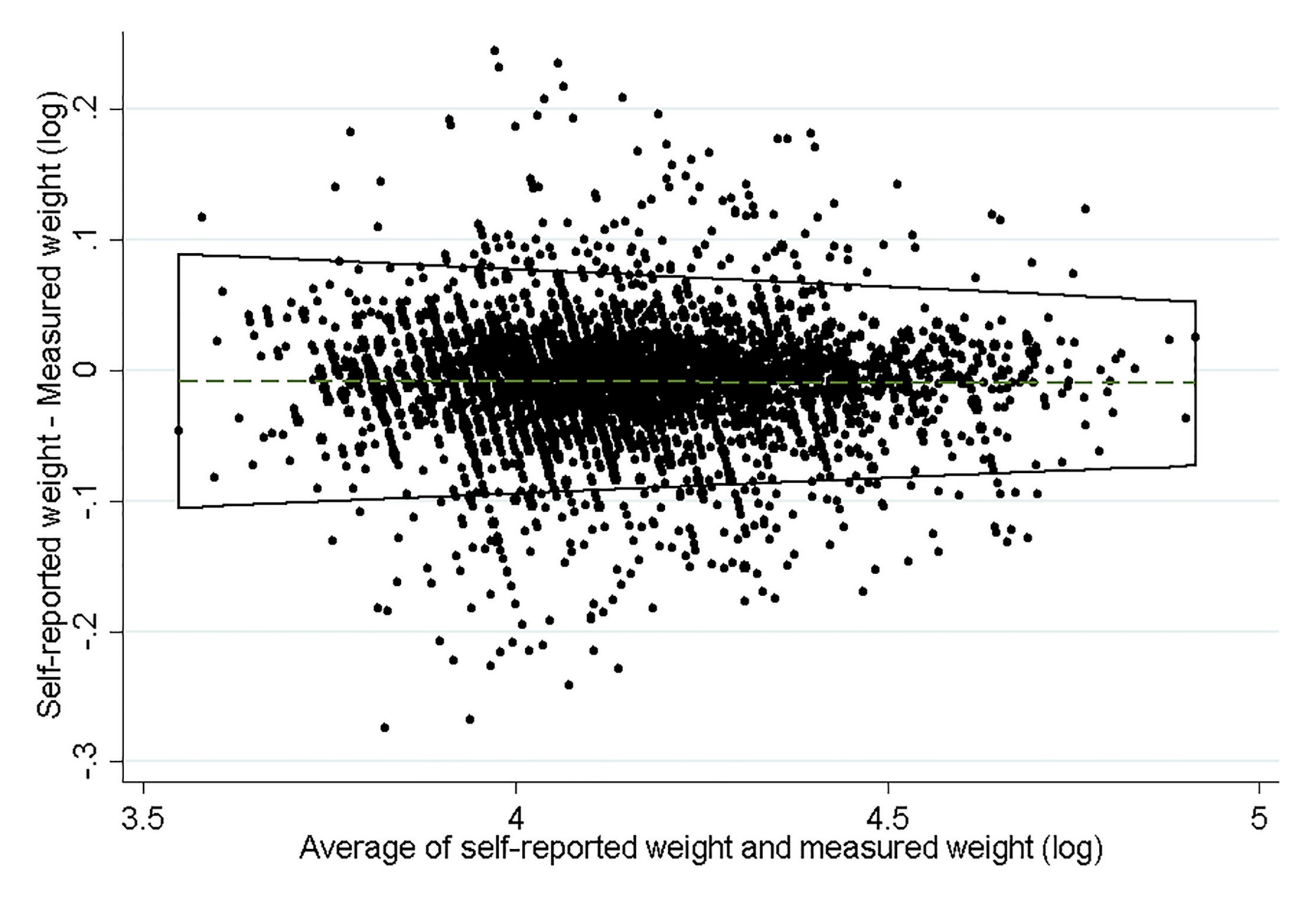
Bland-Altman plot for analysis of agreement between self-reported and measured weight in 18 to 49-year-old women.

Means of self-reported weight and measured weight were 65.7±14.4 kg and 66.3±14.6 kg, respectively. The degree of agreement between log of both variables is displayed in a Bland-Altman plot (Fig 1). The mean difference and limits of agreement increased as the average of weight also did. A higher bias and a variability of weight in women with higher weight were observed. While the amount of data outside the limits of agreement is approximately 9%; within the 95% agreement band in the Bland-Altman plot, there is considerable variability, which could be explained by the different characteristics of each woman.

Mean difference between self-reported weight and measured weight was -0.59±3.21 kg. This difference varied among some sociodemographic characteristics (Table 2). Underreporting of weight increased with increasing category of BMI, from normal weight to obesity.

**Table 2:** Self-reported weight, measured weight, absolute difference and relative difference according to sociodemographic characteristics.

	Self-reported weight (kg)	Measured weight (kg)	Difference (kg)	Relative difference (%)
**Total (*n* = 3,452)**	65.73 ± 14.48	66.31 ± 14.63	-0.59 ± 3.21	-0.76 ± 4.84
**Age**				
18–34 years	62.71 ± 13.57	63.80 ± 14.39	-0.51 ± 3.20	-0.61 ± 4.95
35–49 years	69.57 ± 14.81	70.47 ± 14.61	-0.66 ± 3.21	-0.93 ± 4.69
**Years of schooling**				
≤6 years	66.86 ± 14.90	67.51 ± 14.62	-0.65 ± 3.32	-0.96 ± 4.96
7–9 years	66.88 ± 14.48	67.49 ± 14.69	-0.61 ± 3.28	-0.76 ± 4.85
>9 years	63.76 ± 13.94	64.32 ± 14.35	-0.55 ± 3.00	-0.68 ± 4.59
**Marital status**				
Without a partner	63.07 ± 14.13	63.55 ± 14.51	-0.48 ± 3.16	-0.54 ± 4.84
With a partner	67.24 ± 14.47	67.89 ± 14.45	-0.64 ± 3.23	-0.88 ± 4.83
**Region of residence**				
North^a^	69.66 ±15.62	70.13 ± 15.86	-0.46 ± 3.16	-0.53 ± 4.59
Center^b^	65.06 ± 13.88	65.42 ± 13.94	-0.35 ± 3.10	-0.42 ± 4.66
Mexico City^c^	62.56 ± 15.17	63.71 ± 16.65	-1.14 ± 3.37	-1.30 ± 4.80
South^d^	61.82 ± 12.29	62.85 ± 12.41	-1.02 ± 3.36^a^	-1.50 ± 5.27
**Area of Mexico**				
Rural	65.05 ± 14.10	65.63 ± 14.34	-0.58 ± 3.07	-0.74 ± 4.68
Urban	66.23 ± 14.74	66.82 ± 14.81	-0.59 ± 3.31	-0.77 ± 4.90
**Socioeconomic status**				
Low	63.58 ± 14.02	64.17 ± 14.11	-0.58 ± 3.39	-0.78 ± 5.24
Medium	66.15 ± 14.58	66.82 ± 14.74	-0.67 ± 3.27	-0.88 ± 4.90
High	66.84 ± 14.56	67.35 ± 14.71	-0.51 ± 3.00	-0.65 ± 4.46
**Access to health care**				
Yes	66.41 ± 14.43	66.97 ± 14.64	-0.56 ± 3.00	-0.71 ± 4.49
No	64.36 ± 14.44	64.97 ± 14.44	-0.61 ± 3.61	-0.81 ± 5.51
**Body Mass Index**				
Low weight^a^	43.96 ± 4.17	43.26 ± 4.05	0.70 ± 1.99	1.71 ± 4.69
Normal weight^b^	54.42 ± 6.32	54.51 ± 6.03	-0.09 ± 2.65^a^	-0.11 ± 4.90
Overweight^c^	64.61 ± 7.28	65.31 ± 6.47	-0.69 ± 3.18^a^^b^	-1.08 ± 4.96
Obesity^d^	82.12 ± 12.63	83.26 ± 12.49	-1.13±3.76^a^^b^^c^	-1.31 ± 4.47

Absolute difference = self-reported weight-measured weight; Relative difference = (absolute difference/measured weight) × 100

^a,b,c,d^ different letters indicate significant differences groups each other, p<0.05

In contrast, women with underweight over-reported their weight 0.70±1.99 kg. Compared with women from the Northern region, those from the Southern region showed higher under-reported weight (-0.46±3.16 vs -1.02±3.36 kg). We found a trend of higher under-reported weight in older women, with less years of schooling or living with a partner, when compared with that in women who are younger, more educated, or do not have a partner.

Multiple linear regression models were fitted to relate log-self-reported weight with log-measured weight adjusting for covariates. The final model included those variables with *p* value <0.05 (log-self-reported weight, log-measured height (meters), interaction term (age group × log-self-reported weight), residence in South of Mexico, and living with a partner (married or cohabiting)) with a *R*^2^ = 0.949 (Table 3). We evaluated the goodness of fit in the model using residuals distribution, heteroscedasticity test, and influence measures.

**Table 3:** Regression coefficients for estimating log-weight from log-self-reported weight.

	Β	*p*-value
Constant	0.190	
**Log-self-reported weight**	0.985	< 0.001
**Age group**		
18–34 years	1	
35–49 years	0.179	< 0.001
**Interaction between age group and log-reported weight**	-0.0415	< 0.001
**Log—height (mts)**	0.104	< 0.001
**Region of residence**		
No South	1	
South	0.011	< 0.001
**Marital status**		
Without a partner	1	
With a partner	0.005	0.005
**R**^**2**^	0.9487	
**Residual standard deviation**	**0.048**	

Considering the interaction between age and log-self-reported weight, the resulting model can be expressed as follows:

Women aged 18 to 34 years:

logweightadjusted=0.190+0.985*logselfreportedweight+0.104*logheight(meters)+0.011*RegionSouth+0.005*maritalstatus

Women aged 35 to 49 years:

logweightadjusted=0.190+1.122*logselfreportedweight+0.104*logheight(meters)+0.011*RegionSouth+0.005*maritalstatus

**Fig 2:**
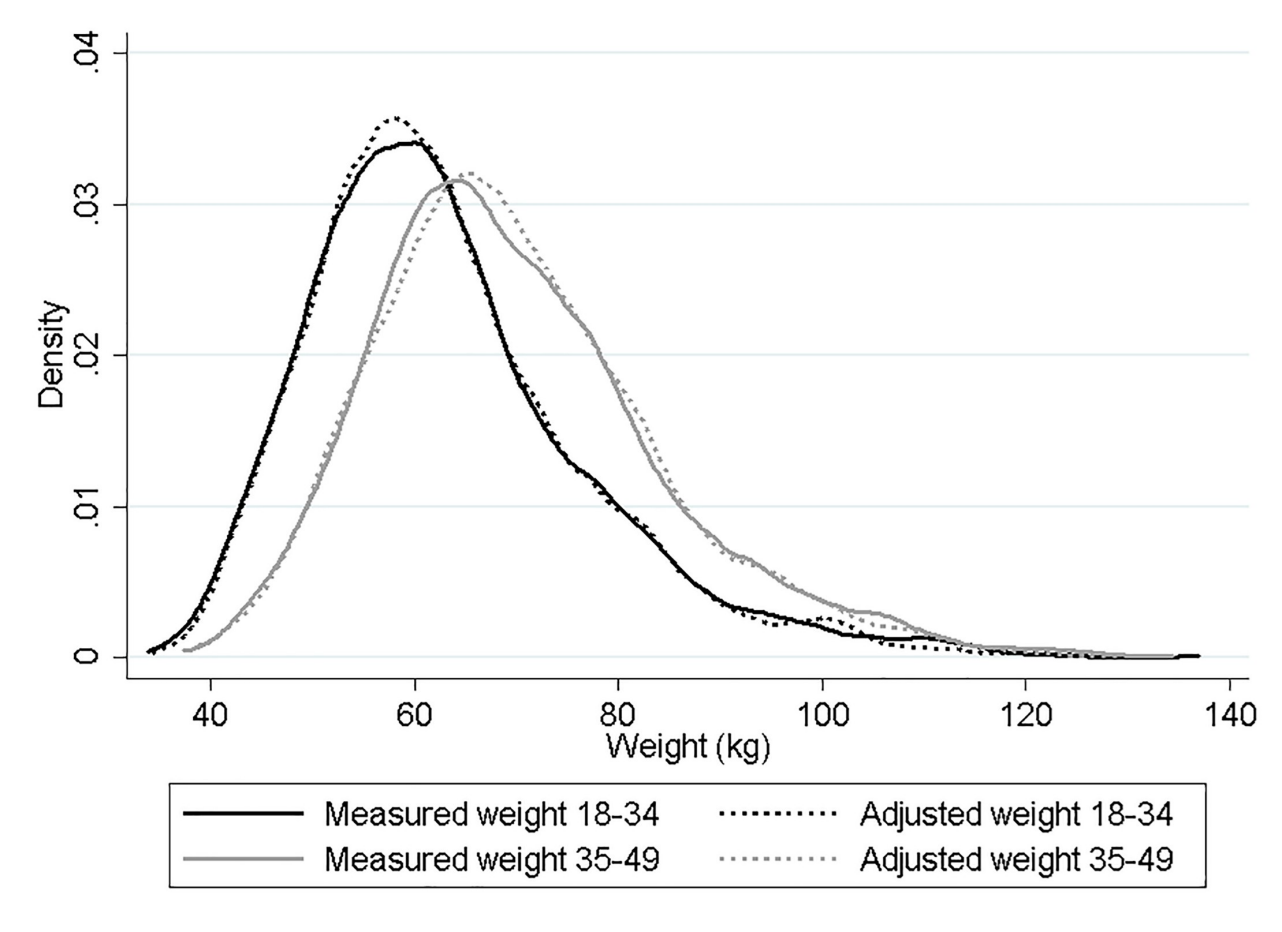
Density distribution of measured weight and adjusted weight by age group.

The result of the model is expressed in logarithm; however, to return to the original scale of the adjusted weight value, the result can be exponentiated. Fig 2 shows adjusted and measured weight distribution by age group. Adjusted weight distribution is similar to measured weight in both age groups. Based on SRW, our findings suggested an overestimation of underweight/normal weight prevalence and an underestimation of overweight or obesity prevalence. The estimation of the prevalence in all BMI categories improved using adjusted weight, where prevalence of overweight or obesity was the same as measured weight in both age groups (Table 4).

**Table 4:** Distribution of BMI based on different versions of weight by age groups.

	BMI calculated using measured weight	BMI calculated using self-reported weight	BMI calculated using adjusted weight
	%	%	%
**18–34 years**			
Low weight	3.5	3.7	3.0
Normal weight	45.9	47.5	46.5
Overweight	31.1	30.0	31.2
Obesity	19.4	18.8	19.3
**35–49 years**			
Low weight	0.52	0.71	0.4
Normal weight	19.1	22.3	18.9
Overweight	40.1	38.0	40.2
Obesity	40.2	38.9	40.4

## Discussion

We found that the agreement between SRW and MW among adult Mexican women of reproductive age differs according to some features. On average, women under-reported their weight (0.5 kg), but differences are significantly higher among women with obesity and among those living in the South of the country. Instead, women with BMI less than 18.5 kg/m^2^ over-reported their weight (0.70 kg).

A recent systematic review that included 21 studies conducted in different countries found that adult women under-reported their weight (-0.94 kg; 95% CI, 1.17–0.71 kg) [19]. In Mexico, two studies recorded a higher mean of under-reported weight (1.41 kg and 1.33 kg) than that from our findings [20, 21]. These slight differences (±1 kg) are expected to be observed, since some intra-variable factors may contribute to misreporting [8]. However, as our findings showed, the latter is not for all women, who may have more extensive variability between the self-reported weight and measured weight.

Differences in the magnitude and variability of under-reporting may result from sociodemographic characteristics [6, 19, 22]. We observed that the difference between SRW and MW was higher in women with obesity than in women with normal weight or overweight. This is consistent with findings derived from studies carried out in Mexico and other countries (United States [23], Japan [24], Sweden [25]). Our results also indicated that women from the South of the country tend to have higher under-reported weight than women from other regions (North, Center, and Mexico City) do. In one study conducted in Mexico, women from Veracruz (state considered in the Southern region of our analysis) had the highest differences between SRW and MW [20]. Furthermore, a systematic review on women of reproductive age shows that the mean difference between SRW and MW was 1.51 kg, 1.14 kg, and 1.02 kg in women from North America, Latin America and the Caribbean, and Europe, respectively [19]. On the other hand, women’s age may affect to what degree women under-report weight, but it is not clear which age groups have the greatest under-reporting [6, 19, 22]. Our results show a trend towards increasing under-reported weight in older adult women, contrary to findings from a Mexican Teachers’ Cohort (MTC), where the mean differences between SRW and MW were 1.8 kg and 1.3 kg in women aged between 30–39 and 45–49 years, respectively [20]. Seijo et al also reported similar results for women of age groups 19–35 and 36–49 years (0.26 kg and 1.05 kg, respectively) [19].

In addition to sociodemographic characteristics, the degree of under-reporting may be due to aspects regarding the time between self-reported weight and direct measurements or the method used to record SRW. The data that we used was collected on the same day and SRW was recorded by personal interview. This may explain the differences of our results compared with other studies carried out in Mexico. In particular, weight measured at MTC was performed 11 months after recording their weight in a self-administered questionnaire [20]. A systematic review shows lower mean differences between SRW and MW when data was collected from an in-person interview and no difference was observed when data was collected on separate days [19].

SRW bias may generate misclassification of BMI and inaccuracy in prevalence estimation of overweight and obesity [5, 8]. Studies had reported a range of underestimation between 0.6% to 11.4% points for overweight prevalence and 0.6% to 11.9% points for obesity prevalence in adult women [26]. This is consistent with our findings where, using SRW to calculate BMI, we observed -1.5% and -0.9% points for overweight and obesity prevalence. Although most of the studies reported that on average women under-reported their weight regardless their BMI, our results indicate that it is the opposite for women with low weight, who tend to over-report.

In women of reproductive age, besides using SRW to classify BMI, accuracy of weight measurement before pregnancy helps to make recommendations about total gestational weight gain (GWG) [1]; therefore, during pregnancy, women with obesity are encouraged to gain less weight (5–9 kg) than women with normal weight (11.5–16 kg) or overweight (7–11.5 kg). Our results indicate that women with obesity tend to have a higher under-reported weight, thus increasing the probability of classifying them with excessive gestational weight gain, because the difference between SRW and MW before pregnancy may scope to -5 kg. In contrast, women with low weight may be prone to be classified as GWG inadequate, since on average they tend to over-report their weight.

Inaccuracy of weight in women of reproductive age may also have an impact on the association with short-term perinatal adverse outcomes. Studies suggest that misclassification of BMI due to weight error bias may have an effect further from the null association [5]. Hence, authors recommend having the most accurate weight, ideally by measuring it, but when this weight is not available, correcting SRW should be considered. Our correction process takes into account those sociodemographic variables that we observed that influence the SRW, and the estimation showed a good improvement on overweight or obesity prevalence in adult women of reproductive age. Other correction procedures have been proposed in a similar population, showing a smaller bias and an increase of the predictive power when the BMI is corrected [10].

In Mexico, there are few data sources that have information on both SRW and MW. National Health Surveys (ENN-99, ENSANUT-2006, ENSANUT 2012, and ENSANUT MC 2016) have data on measured weight but not on self-reported weight. A strength of the present analysis is the use of data from a representative survey, with a large sample size, and measurements of SRW and MW, as well as the inclusion of sociodemographic variables. Nevertheless, because we did not include women without SRW or with difference above 15 kg, our limitation is that our results and the proposed correction process were derived from women with specific characteristics, whose SRW bias is not the greatest, thus there may be other factors that influence the self-reported weight, such as having completed less years of schooling, living in a rural area, and lacking access to health care services. It is also important to note that parity may influence self-reported weight in women [27]. However, we did not include it in the model since it had missing values (75.3%).

## Conclusions

Self-reported weight has limitations to be considered as a direct alternative to measured weight, especially in women of reproductive age with specific characteristics. We suggest that studies using self-reported weight take a correction process into account and assess bias associated with the misreporting. Researchers and Clinicians could use our proposed correction equation to adjust the self-reported weight. However, the latter may not apply to women in other contexts or with specific characteristics.
